# Inhibition of Foxp3 expression in the placenta of mice infected intraperitoneally by *toxoplasma gondii* tachyzoites: insights into the PPARγ/miR-7b-5p/Sp1 signaling pathway

**DOI:** 10.1186/s13071-024-06262-0

**Published:** 2024-04-17

**Authors:** Yue Zhong, Cheng Qin, Qing Wang, Maoyuan Ding, Chong Qiu, Yunzhao Xu, Jinling Chen

**Affiliations:** 1 Department of Pathogen Biology, School of Medicine, Nantong University, 19 Qixiu Road, Nantong, 226001 Jiangsu People’s Republic of China; 2 Department of Obstetrics and Gynecology, Affiliated Hospital of Nantong University, Nantong, 226001 Jiangsu People’s Republic of China

**Keywords:** *Toxoplasma gondii*, microRNA-7b, Forkhead box P3, PPARγ, Sp1

## Abstract

**Background:**

*Toxoplasma gondii*, an obligate intracellular parasitic protozoa, infects approximately 30% of the global population. Contracting *T. gondii* at the primary infection of the mother can result in neonatal microcephaly, chorioretinitis, hydrocephalus, or mortality. Our previous study indicated that pregnant mice infected with *T. gondii* displayed a decrease in both the number and the suppressive ability of regulatory T cells, accompanied by the reduced Forkhead box P3 (Foxp3). Numerous studies have proved that microRNAs (miRNAs) are implicated in *T. gondii* infection, but there is meager evidence on the relationship between alterations of miRNAs and downregulation of Foxp3 induced by *T. gondii*.

**Methods:**

Quantitative reverse transcription polymerase chain reaction was utilized to detect the transcriptions of miRNAs and Foxp3. Protein blotting and immunofluorescence were used to detect the expressions of Foxp3 and related transcription factors. The structure of mouse placenta was observed by hematoxylin and eosin (HE) staining.

To examine the activity of miR-7b promoter and whether miR-7b-5p targets Sp1 to suppress Foxp3 expression, we constructed recombinant plasmids containing the full-length/truncated/mutant miR-7b promoter sequence or wildtype/mutant of Sp1 3' untranslated region (3' UTR) to detect the fluorescence activity in EL4 cells.

**Results:**

In *T. gondii*-infected mice, miR-7b transcription was significantly elevated, while Foxp3 expression was decreased in the placenta. In vitro, miR-7b mimics downregulated Foxp3 expression, whereas its inhibitors significantly upregulated Foxp3 expression. miR-7b promoter activity was elevated upon the stimulation of *T. gondii* antigens, which was mitigated by co-transfection of mutant miR-7b promoter lacking peroxisome proliferator-activated receptor γ (PPARγ) target sites. Additionally, miR-7b mimics diminished Sp1 expression, while miR-7b inhibitors elevated its expression. miR-7b mimics deceased the fluorescence activity of Sp1 3' untranslated region (3' UTR), but it failed to impact the fluorescence activity upon the co-transfection of mutant Sp1 3' UTR lacking miR-7b target site.

**Conclusions:**

*T. gondii* infection and antigens promote miR-7b transcription but inhibit Foxp3 protein and gene levels. *T. gondii* antigens promote miR-7b promoter activity by a PPARγ-dependent mechanism. miR-7b directly binds to Sp1 3' UTR to repress Sp1 expression. Understanding the regulatory functions by which *T. gondii*-induced miR-7b suppresses Foxp3 expression can provide new perspectives for the possible therapeutic avenue of *T. gondii*-induced adverse pregnancy outcomes.

**Graphical Abstract:**

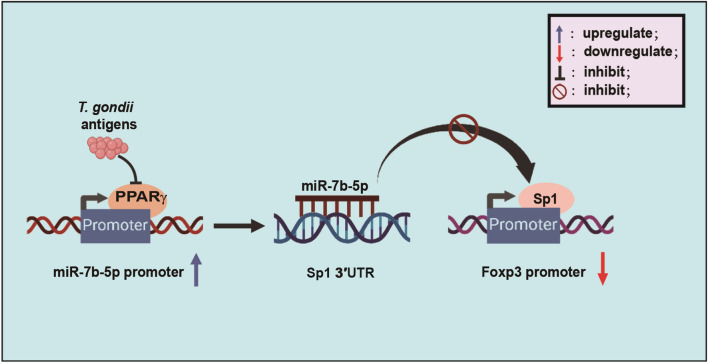

**Supplementary Information:**

The online version contains supplementary material available at 10.1186/s13071-024-06262-0.

## Background

Maternal–fetal immune tolerance is crucial for a successful pregnancy, as it prevents rejection of the allogeneic fetus by the maternal immune system [1]. Early infection with *T. gondii* can induce adverse pregnancy outcome, which is primarily due to the disruption of maternal–fetal immune tolerance rather than the direct invasion of *T. gondii* [2]. Regulatory T (Treg) cells, a unique subset of CD4^+^ T cells, are centrally implicated in establishing and maintaining immune tolerance [3]. The frequency of Treg cells increases in normal human pregnancy, but the frequency or function decreases in pregnancy disorders, such as recurrent pregnancy loss (RPL), preeclampsia (PE), preterm birth (PTB), and recurrent spontaneous abortion (RSA) [4, 5]. Experiments in mice have revealed Treg cell insufficiency in the spleens and placentas of abortion-prone animals. Adoptive transfer of Treg cells from the spleen of healthy pregnant animals can partially prevent fetal rejection and improve pregnancy outcomes, highlighting the crucial role of Treg cells in maintaining healthy pregnancy [6].

microRNAs (miRNAs), belonging to small non-coding RNAs that mainly target the 3' untranslated region (3' UTR) of genes, function as a master driver of translational repression or degradation [7, 8]. miRNAs influence various aspects of immunity, including Treg cell function, homeostasis, and phenotypic stability [9]. Overexpression of miR-17 in vitro and in vivo attenuated the inhibitory activity of Treg cells, which was rescued by co-expression of mutant Eos in the binding sites of Eos and miR-17. Additionally, miR-17 deficiency enhances suppressive function of Treg cells, whereas forced expression of miR-17 confers Treg cell-like Teff cell characteristics, including the production of effector cytokines [10]. miR-125a-deficient Treg cells displayed the reduced function of immune regulation, resulting in more severe pathogenesis of colitis and experimental autoimmune encephalomyelitis [11].

Forkhead box P3 (Foxp3), a specific transcriptional regulator of Treg cells, governs the development, maintenance, and functional maturation of Treg cells [12]. EL4 cells are a T lymphoma cell line that retains many T cell characteristics and is commonly used to study T cell transcriptional regulation. Foxp3 expression can be induced in EL4 cells by anti-CD3/anti-CD28 activation as well as TGF-β stimulation [6]. Treg defects in the decidua of spontaneous abortion mice are associated with reduced levels of the Foxp3 gene and protein [13]. Numerous studies have confirmed the involvement of miRNAs in regulating Foxp3 [14]. Down-regulation of microRNA 182 markedly elevated the percentages of Foxp3^+^ Treg cells in spleen and peripheral lymph nodes. In vivo study confirmed that microRNA 182 levels were negatively correlated with Foxp3 expression [15]. miRNA-221-5p mimics suppressed protein expression of Foxp3 via promoting that of suppressor of cytokine signaling 1 (SOCS1) and receptor-related orphan receptor-gamma-t (RORγt) in mouse model of asthma [16]. Our previous studies have established that the administration of excreted-secreted antigens (ESA) of *T. gondii* can lead to adverse pregnancy outcome in mice, partially due to the number and the suppressive ability of Treg cells that are accompanied by the decreased Foxp3 [17]. Although previous studies have confirmed that miRNAs are critical regulators of Foxp3 expression, whether miRNAs are involved in ESA-induced downregulation of Foxp3 expression remains elusive.

Here, we report that both *T. gondii* and antigens increased miR-7b level but decreased Foxp3 expression. Additionally, our previous study revealed that *T. gondii* antigens affect the promoter activity of Foxp3 via an Sp1-dependent mechanism [18]. *T. gondii* ESA promotes miR-7b promoter activity in a PPARγ-dependent manner, inhibits specific protein 1 (Sp1) expression via targeting Sp1 3' UTR, and thereby represses Foxp3 expression. Taken together, harnessing miR-7b may be a possible therapeutic avenue for adverse pregnancies due to immune imbalances, especially those resulting from *T. gondii* infection.

## Methods

### Mice and mating

ICR (Institute of Cancer Research) mice (6–8 weeks old), obtained from the Animal Research Center of Nantong University, were housed in cages and provided daily access to adequate water and food under appropriate temperature and humidity conditions. The mice were acclimated for 7 days. Female and male mice were paired in a 2:1 ratio and the confirmation of the vaginal plug at 7 a.m. the next morning was regarded as gestational day (gd) 0.5 [19]. Pregnancy mice were randomized into two groups: normal pregnancy and *T. gondii* infection groups. Pregnant mice were injected intraperitoneally with *T. gondii* RH strain tachyzoites (0.5 × 10^3^) on gd 8.5. The animals were sacrificed 10 days post-infection (dpi) with *T. gondii*.

### Preparation and maintenance of* T. gondii* ESA

Tachyzoites of *T. gondii* (RH strain) preserved in liquid nitrogen were revived in ICR mice via intraperitoneal inoculation in our laboratory. *T. gondii* ESA was prepared as previously described [20]. In brief, 1 × 10^7^ tachyzoites were cultured in 6-well plates for 3 h in RPMI medium 1640 (Gibco, Grand Island, NY, USA) without serum and cell supernatants were collected, which was followed by the concentration utilizing Millipore Amicon Ultra-15 centrifugal filter device (Merck Millipore, Darmstadt, Germany). An endotoxin removal kit (Thermo Fisher Scientific, Waltham, MA, USA) was used for endotoxin removal from ESA, following manufacturer’s instructions. The endotoxin level in ESA, assayed by Endotoxin Detection Matrix Limulus Kit (Xiamen Limulus Reagent Co., Ltd., Fujian, China), was less than 0.1 EU/kg. After ESA-containing fluid was filtered by 0.22 μm filters (Merck Millipore), its protein concentration was measured by using the Bradford method.

### Cell culture

The EL4 cell line (Cell Bank of the Shanghai Institute, Chinese Academy of Sciences, China) used in this study was derived from T lymphoma. It is widely used to explore T cell transcriptional regulation because of its retention of T cell properties [21]. In this study, we utilized EL4 cells to study the transcriptional regulation of Foxp3 induced by *T. gondii* ESA, which were cultured in completed Dulbecco’s modified Eagle’s medium (DMEM, Thermo Fisher Scientific) supplemented with fetal bovine serum (10% FBS, ExcellBio, Shanghai, China), streptomycin (100 μg/mL), and penicillin (100 U/mL). EL4 cells were maintained in a 5% CO_2_ humidified environment at 37 ℃ and subcultured every 2 days. EL4 cells were stimulated with ESA (10 μg/mL) for various experiments.

### Cell transfection and electroporation

Table 1 lists the mimics and inhibitors used in this study, including *Mus musculus* (mmu)-miR-7b-5p (miR-7b) inhibitors, miR-7b mimics, mmu-miR-16-5p (miR-16) inhibitors, miR-16 mimics, and corresponding negative controls (NC), which were purchased from Gene Pharma (Shanghai, China). DEPC water was added to 1 OD fragment in tubes following centrifugation at 1400 × g for 1 min following the instructions. miR-7b mimics, miR-7b inhibitors, miR-16 mimics, miR-16 inhibitors, NC mimics, and NC inhibitors were transfected into EL4 cells in 6-well plates at 350 V for 10 ms by using a BTXECM 830 Square Wave Electroporation System (BTX Harvard Apparatus, Holliston, MA, USA). Cells were collected and utilized for western blot (WB) analysis at 48 h post-transfection.

**Table 1:** Primers used in this study

Primer	Sequence (5′ to 3′)
For quantitative real-time PCR	
mmu-miR-7b-5p	TGGAAGACTTGTGATTTTGTTGTT
mmu-miR-16-5p	TAGCAGCACGTAAATATTGGCG
mmu-miR-21a-3p	CAACAGCAGTCGATGGGCTGTC
mmu-miR-27a-3p	TTCACAGTGGCTAAGTTCCGC
mmu-miR-326-3p	CCTCTGGGCCCTTCCTCCAGT
mmu-miR-149-3p	GAGGGAGGGACGGGGGCGGTGC
mmu-miR-185-3p	AGGGGCTGGCTTTCCTCTGGT
mmu-miR-338-3p	TCCAGCATCAGTGATTTTGTTG
mmu-miR-448-3p	TTGCATATGTAGGATGTCCCAT CCCATCCCCAGGAGTCTTG ACCATGACTAGGGGCACTGTA
Foxp3 F	
Foxp3 R	
GAPDH F	AGGTCGGTGTGAACGGATTTG
GAPDH R	TGTAGACCATGTAGTTGAGGTCA
For western blot	
miR-7b mimic F	UGGAAGACUUGUGAUUUUGUUGUU
miR-7b mimic R	CAACAAAAUCACAAGUCUUCCAUU
miR-7b inhibitor F	AACAACAAAAUCACAAGUCUUCCA
miR-16 mimic F	UAGCAGCACGUAAAUAUUGGCG
miR-16 mimic R	CCAAUAUUUACGUGCUGCUAUU
miR-16 inhibitor F	CGCCAAUAUUUACGUGCUGCUA
NC mimic F	UUCUCCGAACGUGUCACGUTT
NC mimic R	ACGUGACACGUUCGGAGAATT
NC inhibitor F	CAGUACUUUUGUGUAGUACAA
For plasmid construction	
WT-Sp1 3′ UTR F	CCGCTCGAGCAACAGAGTGGAAAAGCCTGAAGTA
WT-Sp1 3′ UTR R	ATAAGAATGCGGCCGCATTGTGTGCTTCCCTTTCCCTTAG
MUT-Sp1 3′ UTR F	CGGATTTCAGTATCATTAAAAAAAAAATAGCTATAAGCCTCAATG
MUT-Sp1 3′ UTR R	CATTGAGGCTTATAGCTATTTTTTTTTTAATGATACTGAAATCCG
pGL3-E-7b F	TTACAAGCCTGAGTGTGTGTCCTCCCC
pGL3-E-7b R	GCTAGACACCCTCTCCTGTCCG
pGL3-E-7b-A F	ACTTCATCTTCCCTGTGTAGAATTG
pGL3-E-7b-B F	CTGCAGCTGCTGGTGAGCAGGA
pGL3-E-7b-C F	CTCAAGTTCTTTTTATTTTTAA
pGL3-E-7b-A-Mut 1 F	GCCTTCCCCTTAAGCTTTTTTTTTTTTAACGTCGGAAACTGG
pGL3-E-7b-A-Mut 1 R	CGGAAGGGGAATTCGAAAAAAAAAAAATTGCAGCCTTTGACC
pGL3-E-7b-A-Mut 2 F	TGCCTGTGTCTCAGGTTTTTTTTTTTTAAAGGGATGTCTAGG
pGL3-E-7b-A-Mut 2 R	ACGGACACAGAGTCCAAAAAAAAAAAATTTCCCTACAGATCC

*F* forward, *R* reverse, *miR-7b* microRNA-7b, *WT* wild type, *MUT* mutant, *Sp1* specific protein 1, *3′ UTR* 3′ untranslated region, *pGL3-E-7b* pGL3-Enhancer-miR-7b

### Plasmid construction and transfection

Primers for the full-length sequence and three truncated fragments of the miR-7b promoter (listed in Table 1) were designed on the basis of the miR-7b promoter (GenBank no. NM_ 000083.7, https://www.ncbi.nlm.nih.gov/gene/723883) using the Primer5 (https://www.premierbiosoft.com/primerdesign/) software. PROMO (https://alggen.lsi.upc.es/cgi-bin/promo_v3/promo/promoinit.cgi?dirDB=TF_8.3/) and Jaspar (https://jaspar.genereg.net/) were applied to predict the binding sequence between peroxisome proliferator-activated receptor γ (PPARγ) and miR-7b promoter region, and this prediction was verified by dual luciferase reporter gene experiment. Potential binding sites for the upstream region (2000 bp) of the miR-7b promoter and transcription factor PPARγ were predicted through PROMO and AnimalTFDB (https://guolab.wchscu.cn/AnimalTFDB#!/) databases. The sequences, including the full-length, truncated fragments, and mutants of PPARγ binding sites in the miR-7b promoter region, were then cloned into the pGL3-Enhancer vector (pGL3-E) to construct pGL3-Enhancer-miR-7b (pGL3-E-7b), pGL3-E-7b-A/B/C, pGL3-E-7b-A-Mut 1/Mut 2, and pGL3-E-7b-A-Mut 1 + 2, respectively. Primers for the Sp1 3′ UTR (GenBank accession no. NM_ 013672, https://mirdb.org/cgi-bin/target_detail.cgi?targetID=2598711) were designed using Primer5 software, incorporating the *Xho*I (New England Biolabs, lpswich, MA, USA) and *Not*I (New England Biolabs) restriction sites (listed in Table 1). The targeted binding sites of miR-7b and Sp1 3′ UTR were predicted via miRDB (http://mirdb.org) and TargetScan (http://www.targetscan.org/vert_72/). The Sp1 3′ UTR mutant was generated using the primers listed in Table 1 through overlap PCR. The sequences, including the wild-type (WT) or mutant (MUT) of Sp1 3′ UTR, were cloned into psiCHECK-2 vectors. Recombinant plasmids, with or without the Renilla luciferase plasmid (pRL-TK), were electroporated into EL4 cells, which were subsequently treated with ESA at 24 h post-electroporation and cultured for an additional 24 h.

### Luciferase reporter assay

EL4 cells (1 × 10^6^), seeded in a 6-well plate, were co-transfected with pGL3-E-7b, pGL3-E-7b-A/B/C, pGL3-E-7b-A-Mut 1/Mut 2, pGL3-E-7b-A-Mut 1 + 2, and the Renilla luciferase plasmid (pRL-TK). In some experiments, EL4 cells (1 × 10^6^) were transfected with recombinant plasmids containing the WT or MUT form of Sp1 3′ UTR with miR-7b mimics/NC mimics or miR-7b inhibitors/NC inhibitors. The cultured cells were lysed and centrifuged at 12,400 × *g* for 5 min 48 h post-electroporation. The supernatant was collected for luciferase activity analysis by luciferase assay kit (Promega, Madison, WI, USA), which measures the ratio of firefly luciferase to Renilla luciferase.

### Western blot analysis

Total proteins that were extracted from EL4 cells with RIPA buffer containing phosphatase inhibitor as well as protease inhibitor were separated in 10% sodium dodecyl sulfate–polyacrylamide gel electrophoresis (SDS-PAGE) and were subsequently transferred to polyvinylidene fluoride (PVDF) membranes (Merck Millipore). After PVDF membrane was blocked with 5% nonfat milk for 2 h, it was sequentially incubated overnight at 4 ℃ with the following primary antibodies: PPARγ (CST, Danvers, MA, USA), ELK1 (CST), ETS1 (Proteintech, Rosemont, USA), Foxp3 (Abcam, Cambridge, UK), and Sp1 (Abcam). Horseradish peroxidase (HRP)-labeled goat anti-mouse antibody (Proteintech) or HRP-labeled goat anti-rabbit antibody (Proteintech) was utilized as the secondary antibody [22]. The proteins were subsequently revealed with enhanced chemiluminescence (ECL, Meilunbio, Dalian, China). The density of the bands (versus GAPDH) was analyzed using ImageJ software (National Institutes of Health, Bethesda, MD, USA).

### RNA extraction, retro-transcription, and quantitative real-time PCR

Small RNA, isolated from mouse placentas as well as EL4 cells through RNAiso for Small RNA (Takara, Kyoto, Japan), was reversely transcribed with the Mir-X miRNA First-Strand Synthesis Kit (Takara). Total RNA was extracted from EL4 cells with Trizol (Invitrogen, Waltham, CA, USA), and synthesized into cDNA using a RevertAid First Strand cDNA Synthesis Kit (Thermo Fisher Scientific). The primer sequences including miR-7b, miR-16, miR-21a, miR-27a, miR-326, miR-149, miR-185, miR-338, miR-448, Foxp3, and GAPDH are listed in Table 1. The polymerase chain reaction (PCR) was then performed on a StepOne^™^ real-time fluorescent quantitative PCR instrument (Applied Biosystems, Foster City, CA, USA) by using the SYBR Premix Ex Taq RT-PCR Kit (Takara) in accordance with the manufacturer′s protocol. PCR reactions were performed under the following conditions: initial denaturation at 95 ℃ for 30 s, followed by denaturation at 95 ℃ for 5 s, annealing at 62 ℃ for 30 s, 40 cycles at 72 ℃ for 30 s, and a final extension at 95 ℃ for 15 s, 62 ℃ for 30 s, and 95 ℃ for 15 s. miRNA abundance was determined using the comparative cycle time (Ct) method compared with U6, an internal control for RNA normalization. Relative miRNA levels were quantified with the 2^−△△Ct^ method.

### HE staining

Mouse placental tissue was fixed with 4% paraformaldehyde, dehydrated in ethanol (80%, 90%, 100%, respectively) and sequentially embedded in paraffin were serially sectioned at 5 μm. Then, each section was dewaxed in xylene, sequentially dehydrated in graded ethanol, stained with hematoxylin solution for 5 min, differentiated in hydrochloric acid ethanol (1%), stained with eosin (1%) for 2 min, subsequently dehydrated in graded ethanol, permeabilized with xylene, and mounted with neutral gum. Images were captured using a Leica DM 5000 B microscope (Leica Biosystems, Wetzlar, Germany).

### Immunofluorescence staining

EL4 cells seeded in 6-well plates were fixed with paraformaldehyde (4%) for 2 h. Next, each sample was washed thrice with PBS for 5 min, centrifuged to discard the supernatant, resuspended on a polylysine-coated glass slide, dried in a fume hood, and incubated in a solution containing 0.5% Triton X-100 and 5% bovine serum albumin. Cells were then incubated respectively with primary antibody overnight at 4 ℃. After washing with PBS, the sample was subsequently incubated with Alexa Fluor 488-conjugated or Alexa Fluor 568-conjugated secondary antibodies (Invitrogen) at room temperature for 30 min in the dark. Nuclei were stained with Hoechst 33,342 following PBS washing. Final image was acquired using the Leica fluorescence microscope (magnification × 40).

### Statistical analysis

All experiments in vivo and in vitro were performed at least three times. The data, expressed as mean ± standard deviation (SD), were analyzed by using GraphPad Prism 5 (GraphPad Inc, La Jolla, CA, USA). *P* < 0.05 was considered a significant difference between different conditions, determined by a two-tailed unpaired *t* test, one-way analysis of variance (ANOVA) with Tukey’s multiple comparisons test or two-way ANOVA with Sidak’s multiple comparisons test as indicated in the figure legends.

## Results

### *T. gondii* infection affected some related microRNA transcription and Foxp3 expression in mouse placenta

**Fig. 1:**
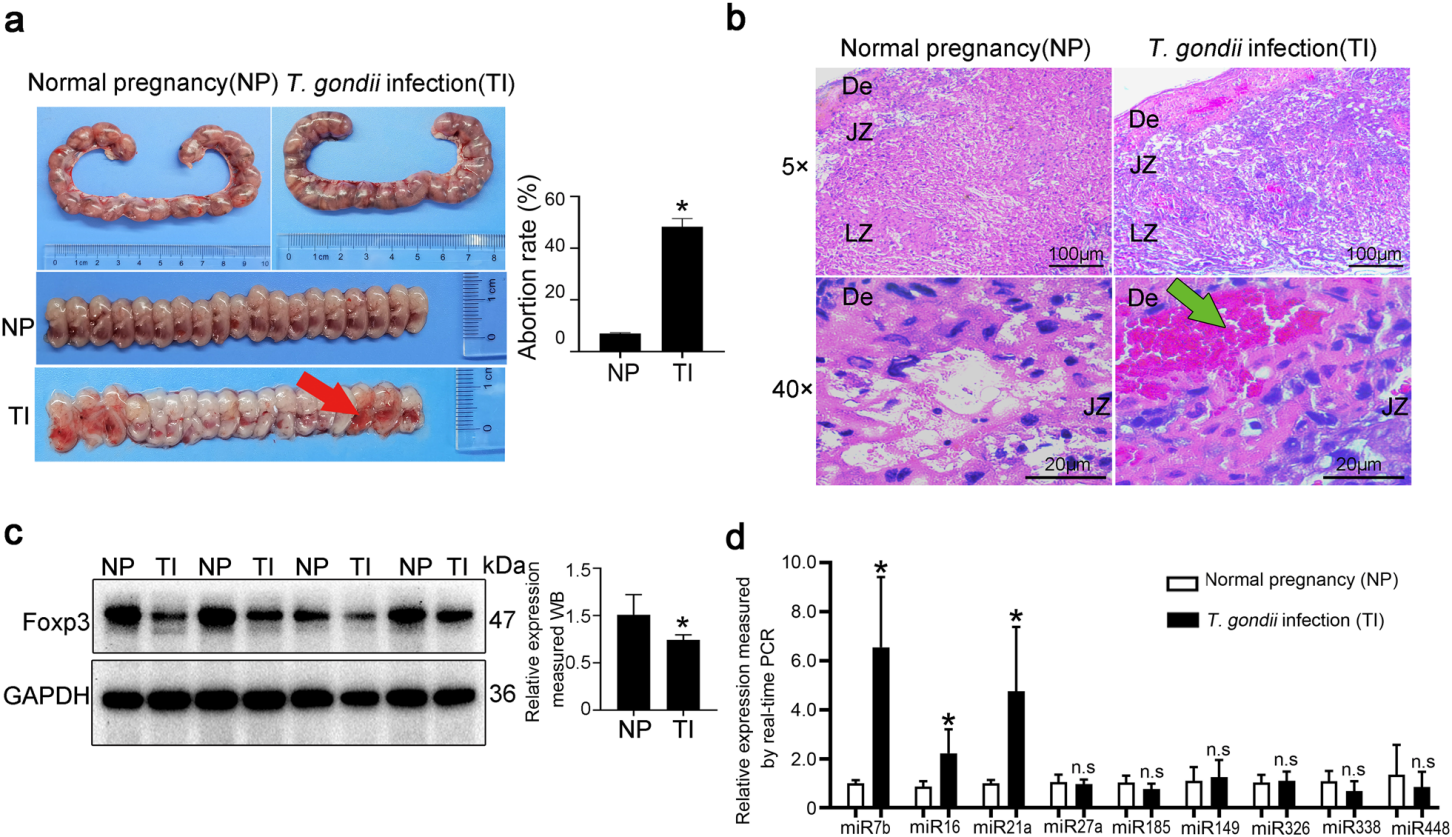
Abnormal pregnancy caused by *T. gondii* infection in mice was associated with Foxp3 and miRNAs. **a** Images are representative photographs of the uterus and fetus on 10 dpi and the abortion rate was analyzed (*n* = 4–5 mice). Compared with normal pregnancy in mice, obvious hemorrhage and necrosis in the mouse placenta along with fetal miscarriage were observed in the infected pregnant group (red arrow). **b** Pathological alterations of mouse placentas, which are composed of De, JZ and LZ, were observed by HE staining (*n* = 4–5 mice). A distinct placental hemorrhage occurred in the infected group (green arrow). **c** Foxp3 expression in the mouse placenta was measured by western blot and analyzed by Image J, normalized to GAPDH (*n* = 4 mice). **d** miRNAs in the placenta of mice with *T. gondii* (RH strain) infection were measured by real-time PCR, which is normalized to U6 (*n* = 4–5 mice). NP: normal pregnant mice; TI: *Toxoplasma*-infected pregnant mice. Data were presented as mean ± SD. Statistical analysis was conducted using two-tailed unpaired Student’s *t*-test (**a**, **c**, and **d**). *: *P* < 0.05; n.s.: *P* > 0.05

After the infection of *T. gondii*, the pregnant mice exhibited symptoms such as sluggish responses, for example, piloerection, distinct trembling, and uncoordinated walking. At gd 18.5, normal pregnant mice showed well-developed fetuses and placentas. However, the RH-infected group exhibited delayed fetal development and reduced embryo size. In parallel, the abortion rate in the infected group was prominently higher than that of uninfected mice (Fig. 1a). Placental structures from normal and RH-infected pregnant mice were examined using HE staining. Placenta of normal mouse is composed of the decidual zone (De), junction zone (JZ), and labyrinth zone (LZ). In the RH-infected group, significant hemorrhage was observed in the JZ area of the placenta, indicating a potential decrease in nutrient supply and impaired gas exchange (Fig. 1b). Together these results confirm that *T. gondii* infection during pregnancy leads to structure changes in the maternal–fetal interface of mice.

Our data manifested that Foxp3 expression was markedly reduced in the placentas of mice infected with *T. gondii* compared with that in uninfected mice (Fig. 1c). In an attempt to explore whether miRNAs are implicated in governing Foxp3 expression, several miRNAs (miR-7b, miR-16, miR-21a, miR-27a, miR-185, miR-149, miR-326, miR-388, and miR-448) were screened and validated using real-time PCR. Comparing *T. gondii*-infected and uninfected samples revealed that three of the nine miRNAs were significantly elevated (miR-7b, miR-16, and miR-21a) while the remaining six (miR-27a, miR-185, miR-149, miR-326, miR-388, and miR-448) did not exhibit any significant difference at the transcription levels (Fig. 1d). These results suggest a potential link between *T. gondii* infection in mice and adverse pregnancy outcomes characterized by reduced Foxp3 levels and increased miRNA expression.

### *T*.* gondii* antigens induce microRNA-7b and microRNA-16 transcriptions in vitro

**Fig.2:**
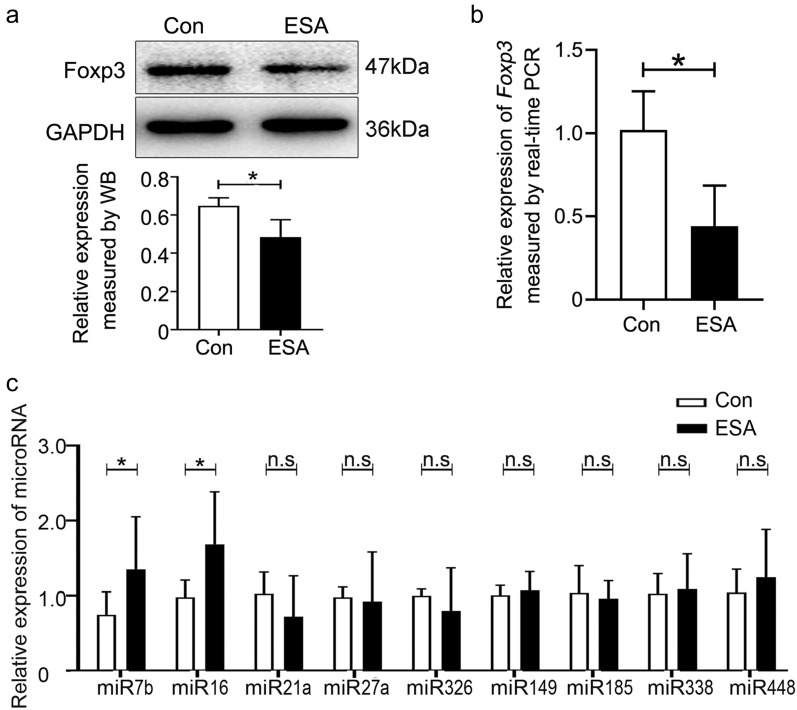
*T. gondii* antigens downregulated Foxp3 but upregulated miRNA-7b and miRNA-16 in vitro. **a** Foxp3 expression in EL4 cells stimulated by ESA for 24 h was assayed by western blot. **b** mRNA level of Foxp3 in EL4 cells stimulated by ESA for 12 h was determined by real-time PCR. **c** microRNAs in EL4 cells at 12 h post-stimulation of ESA were assayed by real-time PCR. Con: Control; ESA: *T. gondii* excreted-secreted antigens; Data were representative of the results of three independent experiments (mean ± SD). Statistical analysis was conducted using two-tailed unpaired Student’s *t*-test. *: *P* < 0.05; n.s.: *P* > 0.05

To investigate the possible relationship between Foxp3 and its associated miRNAs in vitro, we used EL4 cells to establish a cell model expressing Foxp3. To further ascertain the regulatory effect of *T. gondii* antigens on Foxp3 expression, 10 μg/mL ESA (Additional file1: Fig. S1) was applied to stimulate EL4 cells for 24 h. Consistent with the aforementioned in vivo findings, both protein and gene levels of Foxp3 were significantly reduced in *T. gondii* ESA-stimulated EL4 cells compared with those in the control group (Fig. 2a, b). The transcription levels of the predicted miRNAs were validated using real-time PCR. The validation results indicated a significant upregulation of miR-7b and miR-16 in the ESA-stimulated group. However, there was no statistical difference in the transcription levels of miR-21a, miR-27a, miR-185, miR-149, miR-326, miR-388, and miR-448 in EL4 cells compared with those in the control group (Fig. 2c). These data suggested a potential association between Foxp3 downregulation and miR-7b and miR-16 expression in ESA-stimulated EL4 cells in vitro*.*

### microRNA-7b inversely regulates Foxp3 expression

**Fig. 3:**
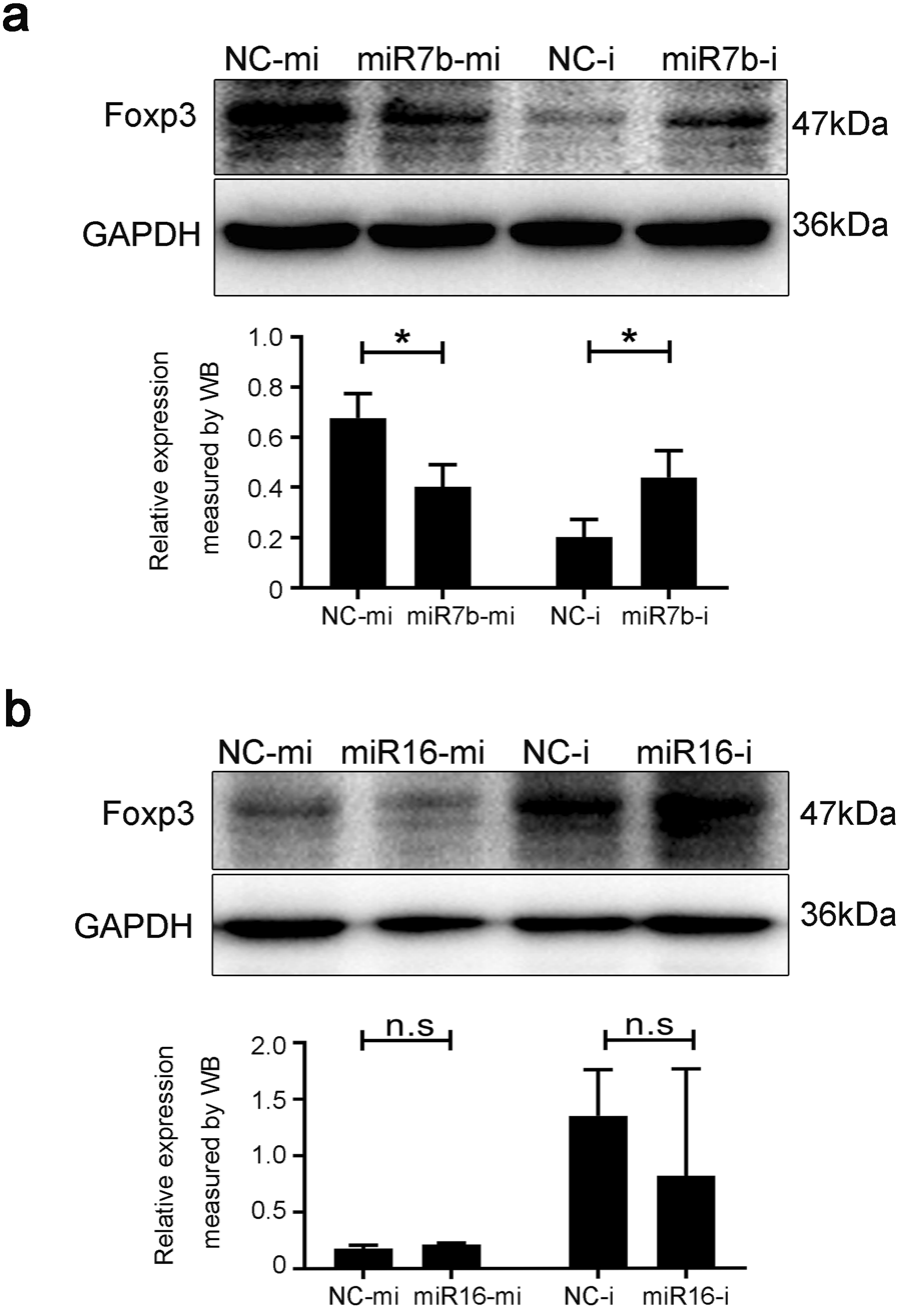
miR-7b inhibited Foxp3 expression. **a** Foxp3 expression was assessed by using western blot in EL4 cells that were transfected with mimics or inhibitors (NC-mi, miR-7b-mi, NC-i, and miR-7b-i), respectively. NC-mi: negative controls of miRNA mimics; miR-7b-mi: microRNA-7b mimics; NC-i: negative controls of miRNA inhibitors. miR-7b-i: microRNA-7b inhibitors. **b** Foxp3 expression was detected by using western blot in EL4 cells transfected with mimics or inhibitors (NC-mi, NC-i, miR-16-mi, and miR-16-i), respectively. miR-16-mi: microRNA-16 mimics; miR-16-i: microRNA-16 inhibitors. Data were representative of the results of three independent experiments (mean ± SD). Statistical analysis was conducted using two-way ANOVA with Sidak’s multiple comparisons test (a and b). *: *P* < 0.05. n.s.: *P* > 0.05

To further confirm the potential link between decreased Foxp3 and increased miR-7b and miR-16 levels in vitro, we performed a transfection experiment using miR-7b and miR-16 mimics or inhibitors in EL4 cells. Western blot analysis demonstrated in Fig. 3a and b that Foxp3 expression was prominently diminished in the group of miR-7b mimics but observably enhanced in the group of miR-7b inhibitors. However, there was no alteration in the miR-16 mimic or inhibitor groups compared with the corresponding negative controls. This highlights the inverse correlation between miR-7b and Foxp3. These data indicated that miR-7b might emerge as a negative regulator of Foxp3 expression.

### *T. gondii* antigens enhance promoter activity of microRNA-7b

**Fig. 4:**
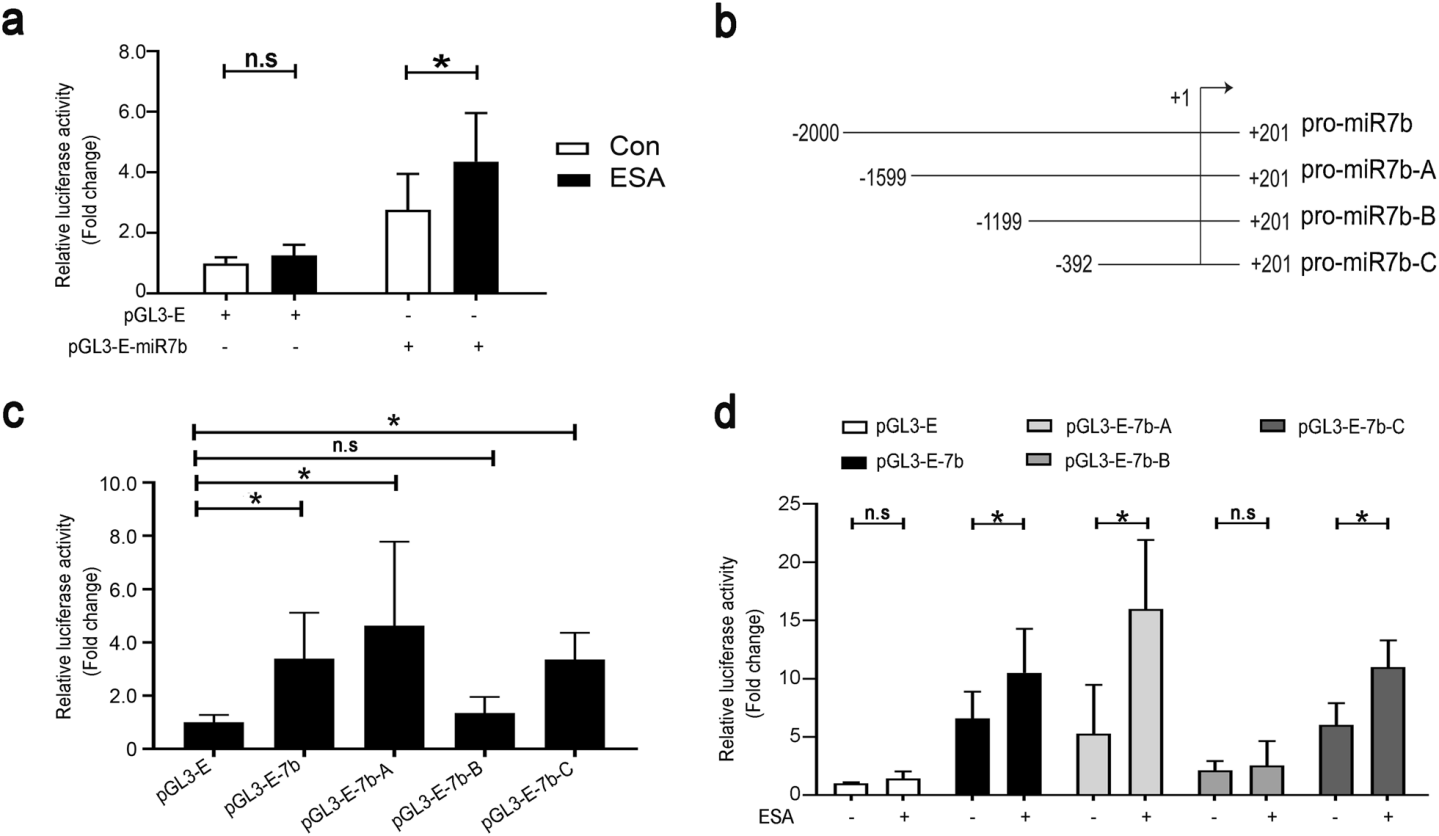
*T. gondii* antigens elevated the miR-7b promoter activity. **a** Luciferase reporter assay was applied to detect the luciferase activity in EL4 cells that were electroporated with pGL3-E or pGL3-E-7b and then stimulated by ESA for 24 h, respectively. **b** Schematic diagram showed the truncation sequences at miR-7b promoter region. **c** The luciferase activities in EL4 cells, transfected respectively with recombinant plasmids, including pGL3-E, pGL3-E-7b, and pGL3-E-7b-A/B/C, were evaluated at 48 h post-transfection. **d** The luciferase activities in EL4 cells that were transfected respectively with recombinant plasmids including pGL3-E, pGL3-E-7b, and pGL3-E-7b-A/B/C, and then stimulated by ESA for 24 h and assayed by luciferase reporter assay at 48 h post-transfection. Data were representative of the results of three independent experiments (mean ± SD). Statistical analysis was conducted using one-way ANOVA Tukey’s multiple comparisons test (**c**) and two-way ANOVA with Sidak’s multiple comparisons test (**a** and **d**). *: *P* < 0.05. n.s.: *P* > 0.05

To analyze the possible mechanism by which *T. gondii* antigens enhanced miR-7b transcription, we examined the promoter activity of miR-7b. Initially, we constructed pGL3-E-7b plasmids containing the complete miR-7b promoter sequence. Subsequently, we used a dual-luciferase system to validate the miR-7b promoter activity in ESA-treated EL4 cells. As shown in Fig. 4a, luciferase reporter analysis revealed that ESA treatment significantly enhanced miR-7b promoter activity by approximately threefold in EL4 cells electroporated with pGL3-E-7b, but had no effect on the pGL3-E group. To identify the regulatory mechanism of miR-7b transcription, three truncated fragments of the miR-7b promoter were inserted into pGL3-Enhancer plasmids to establish recombinant plasmids, named pGL3-E-7b-A (−1599/+201), pGL3-E-7b-B (−1199/+201), and pGL3-E-7b-C (−392/+201) (Fig. 4b). Truncated promoter activity analysis revealed a significant increase in the promoter activity of EL4 cells transfected with plasmids containing the −1599/+201 and −392/+201 regions of the miR-7b promoter, compared with that in the pGL3-E group. There was no significant difference in promoter activity between the pGL3-E-7b-B and pGL3-E transfection groups. This suggested that the active regions of the miR-7b promoter were indeed located in the −1599/−1199 and −392/+201 regions (Fig. 4c). Additionally, EL4 cells were treated with ESA to verify the luciferase activity of all the truncated fragments of the miR-7b promoter. ESA administration significantly increased the luciferase activity in EL4 cells electroporated with pGL3-E-7b, pGL3-E-7b-A, or pGL3-E-7b-C, but failed to elevate its luciferase activity upon the transfection of pGL3-E or pGL3-E-7b-B (Fig. 4d). These findings suggested that ESA enhances miR-7b promoter activity in EL4 cells by targeting the −1599/−1199 and −392/+201 regions of the miR-7b promoter. These regions may contain potential transcription factor (TF)-responsive elements that contribute to increased promoter activity of miR-7b.

### PPARγ targets the miR-7b promoter region

**Fig. 5:**
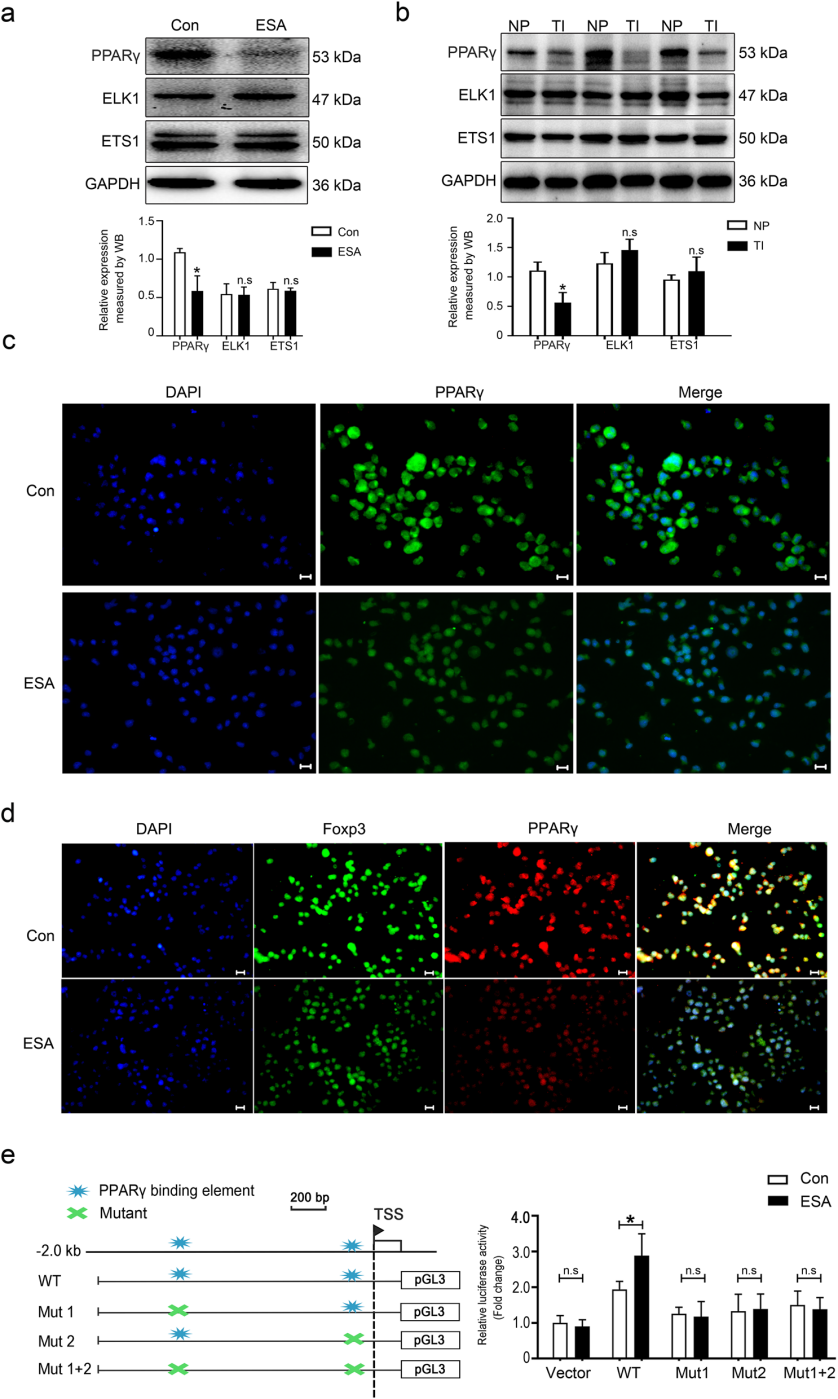
PPARγ bound to the region of miR-7b promoter. **a** Western blot was conducted to validate the expressions of PPARγ, ELK1, and ETS1 in EL4 cells stimulated by ESA for 24 h in vitro. **b** The expressions of PPARγ, ELK1, and ETS1 in mouse placentas infected with *T. gondii* were assayed by western blot (*n* = 3 mice). **c** PPARγ expression in EL4 cells stimulated by ESA for 24 h in vitro was detected by immunofluorescence. Scale: 40 μm. **d** The expression of Foxp3 and PPARγ in EL4 cells stimulated with ESA in vitro for 24 h was detected by immunofluorescence double staining. Nuclear: DAPI, blue; Foxp3: green; PPARγ: red. Scale: 40 μm. **e** The luciferase activity in EL4 cells that were transfected respectively with recombinant plasmids containing the WT or mutations (Mut 1, Mut 2, and Mut 1 + 2) sequences of miR-7b promoter was detected by luciferase reporter assay at 48 h post-transfection. NP: normal pregnant mice; TI: *Toxoplasma*-infected pregnant mice; ESA: *T. gondii* excreted-secreted antigens; TSS: transcriptional starting site. Data were representative of the results of three independent experiments (mean ± SD). Statistical analysis was conducted using two-tailed unpaired Student’s *t*-test. *: *P* < 0.05; n.s.: *P* > 0.05

Potential TFs within the miR-7b promoter region were predicted and analyzed using PROMO and Jaspar. We utilized WB to confirm the effect of *T. gondii* and its antigens on the involved potential TFs. Western blot analysis revealed that ESA markedly inhibited PPARγ expression in vitro, but had no effect on the expression of E26 transformation-specific-1 (ETS1) and Ets-like domain-containing protein 1 (ELK1) (Fig. 5a). Similarly, the placentas of *Toxoplasma*-infected mice exhibited a comparable phenotype (Fig. 5b). PPARγ expression in ESA-treated EL4 cells was detected using immunofluorescence, and the results were in line with the expression in vitro and in vivo as discussed previously (Fig. 5c). Double immunofluorescence staining was performed to investigate the potential colocalization of Foxp3 and PPARγ. Cytofluorimetric results indicated the colocalization of Foxp3 and PPARγ in EL4 cells. Additionally, the fluorescence intensities of both Foxp3 and PPARγ decreased following ESA stimulation (Fig. 5d).

We analyzed the binding sites within the upstream region (2000 bp) of the miR-7b promoter and PPARγ using the PROMO and AnimalTFDB databases. The results revealed that PPARγ had two potential binding sites (nucleotides −1242/−1231 and −128/−117) on the miR-7b 5′-flank promoter, which is consistent with the analysis of promoter activity in EL4 cells transfected with plasmids containing truncated fragments of the miR-7b promoter. Next, we mutated one or both of them to determine the functional site. ESA administration increased luciferase activity in EL4 cells transfected with pGL3-E-7b-A but had no effect on luciferase activity in EL4 cells transfected with pGL3-E-7b-A-Mut 1, pGL3-E-7b-A-Mut 2, or pGL3-E-7b-A-Mut 1 + 2 (Fig. 5e). These findings indicated that treatment with *T. gondii* antigens led to a notable increase in promoter activity of miR-7b. This increase was dependent on PPARγ, which binds to specific regions of the miR-7b promoter (−1242/−1231 and −128/−117).

### miR-7b suppresses Sp1 expression by targeting its 3′ UTR

**Fig. 6:**
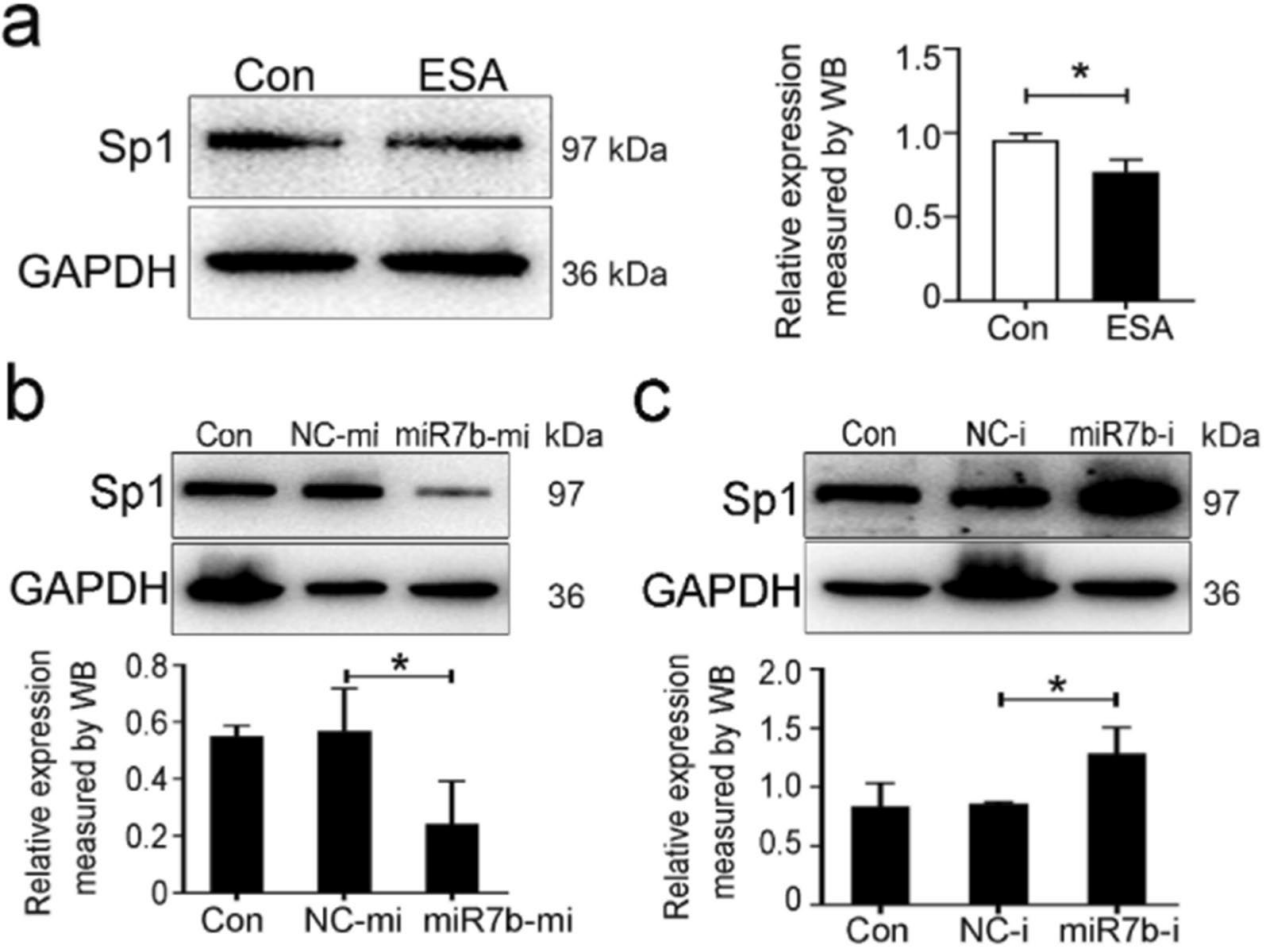
miR-7b suppressed Sp1 expression. **a** Sp1 expression was assessed by using western blot analysis in EL4 cells stimulated by ESA for 24 h in vitro. **b** Sp1 expression was detected by using western blot after 48 h-transfection in EL4 cells that were transfected with NC-mi or miR-7b-mi, respectively. NC-mi: negative controls of miRNA mimics; miR-7b-mi: microRNA-7b mimics. **c** Sp1 expression was detected by using western blot in EL4 cells that were transfected with NC-i or miR-7b-i. NC-i: negative controls of miRNA inhibitors; miR-7b-i: microRNA-7b inhibitors; ESA: *T. gondii* excreted-secreted antigens. Data were representative of the results of three independent experiments (mean ± SD). Statistical analysis was conducted using two-tailed unpaired Student’s *t*-test (**a**), or one-way ANOVA Tukey’s multiple comparisons test (**b** and **c**). *: *P* < 0.05. n.s.: *P* > 0.05

Bioinformatics analysis using TargetScan and miRDB software revealed that miR-7b could bind to the 3′ UTR of Sp1. To explore the effect of miR-7b on governing Sp1 expression, ESA (10 μg/mL) was applied to stimulate EL4 cells for 24 h. The inhibitory effect of the *T. gondii* antigen on Sp1 expression was verified using WB analysis (Fig. 6a). To further explore the correlation between miR-7b upregulation and Sp1 downregulation, WB was applied to evaluate Sp1 expression in EL4 cells that were transfected with miR-7b mimics, inhibitors, or the corresponding negative controls. As shown in Fig. 6b and c, Sp1 expression in EL4 cells with the transfection of miR-7b mimics was prominently lower than that in the mimic control group, whereas Sp1 expression in EL4 cells with the transfection of miR-7b inhibitors was strikingly higher than that in the inhibitor control group. Consequently, miR-7b expression negatively correlated with Sp1 expression in vitro.

**Fig. 7:**
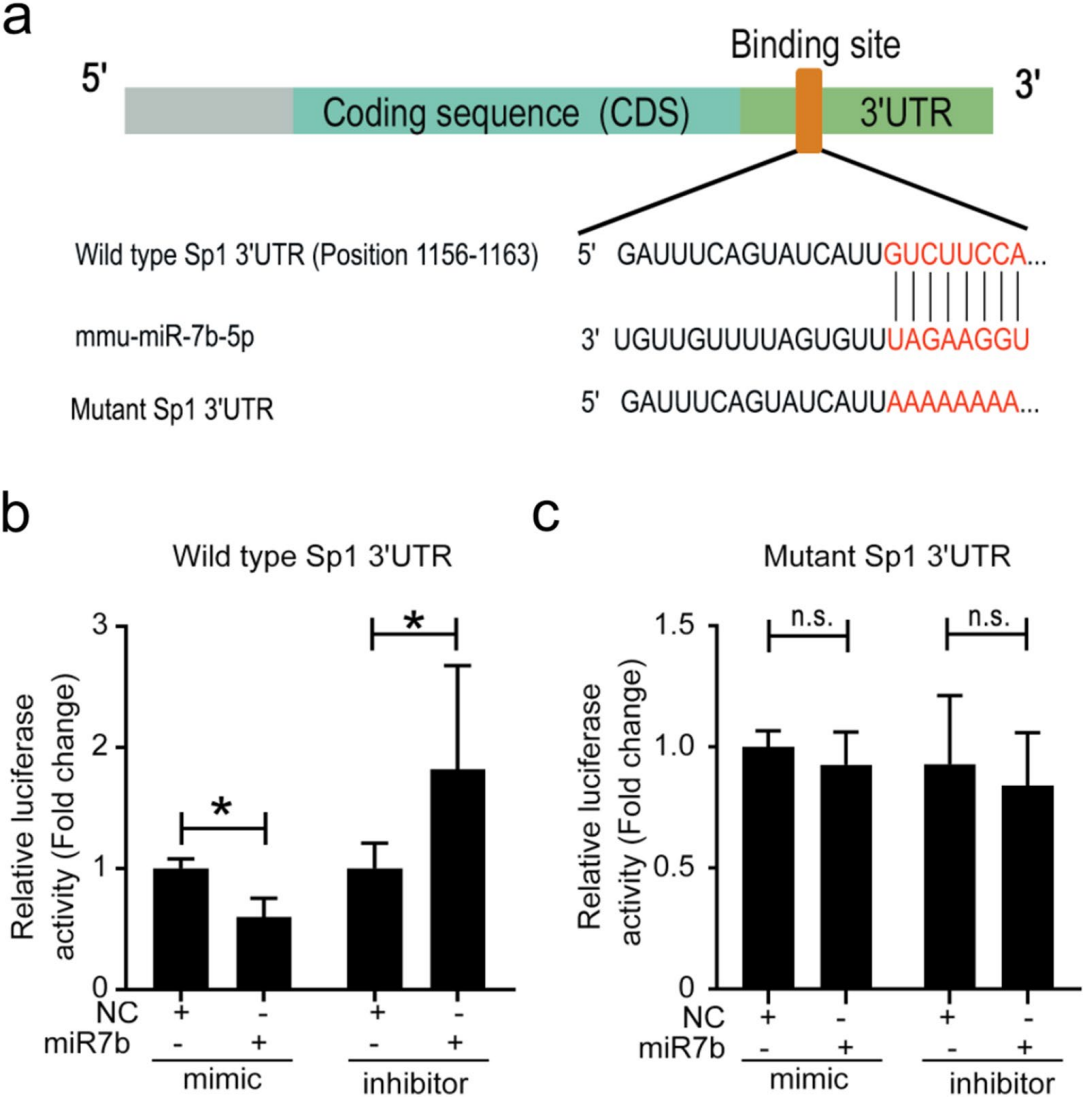
miR-7b bound to Sp1 3' UTR. **a** Schematic representation was shown for predicted binding sites of miR-7b and Sp1 3' UTR on the basis of the analysis of bioinformatics. The binding sites of miR-7b and Sp1 3' UTR were point mutated to construct the mutant plasmids. **b**, **c** The luciferase activities in EL4 cells that were co-transfected with recombinant plasmids containing the WT or MUT of Sp1 3' UTR and miR-7b mimics or inhibitors were assayed at 48 h post-transfection. Data were representative of the results of three independent experiments (mean ± SD). Statistical analysis was conducted using two-way ANOVA with Sidak’s multiple comparisons test (**b** and **c**). *: *P* < 0.05. n.s.: *P* > 0.05

Next, we elucidated the mechanism by which miR-7b inhibited Sp1 expression. TargetScan analysis predicted that miR-7b may target Sp1 on the basis of the matching of the miR-7b seed sequence region to the 3′ UTR sequence of Sp1 (1156–1163) (Fig. 7a). To clarify the direct interaction between miR-7b and Sp1, plasmids containing WT or MUT Sp1 3′ UTR sequences were constructed. As shown in Fig. 7b and c, miR-7b mimics significantly inhibited luciferase activity in EL4 cells that were transfected with the recombinant plasmid containing WT Sp1 3′ UTR, but failed to suppress its activity after transfection with MUT Sp1 3′ UTR. Collectively, these data indicate that miR-7b represses Sp1 expression by directly targeting its 3′ UTR.

## Discussion

Contracting *T. gondii* during pregnancy can lead to severe complications such as abortion, stillbirth, and fetal teratogenicity [23, 24]. *T. gondii* infection-induced adverse outcomes are primarily correlated to the disruption of immune tolerance rather than the replication of *T. gondii* [25]. ESA was often used to explore the mechanism of maternal–fetal immune imbalance in in vivo and in vitro studies [6, 18, 26]. *T. gondii* lysate antigens (TLA) do trigger the immune response, which induces increased levels of IL-10 and TGF-β in BV-2 microglial cultured in vitro [27]*.* Nonetheless, it is also pointed out that phagocytosis of parasites is insufficient to trigger *T. gondii*-specific CD4^+^ and CD8^+^ T cell responses [28]. Conversely, CD4^+^ tachyzoite-specific T cell clones, derived from three chronically infected healthy subjects, proliferated in response to ESA, suggesting the shared immunodominant antigens between *T. gondii* tachyzoites and ESA [29]. Additionally, *T. gondii* is recruited to the parasitophorous vacuole membrane for its survival and replication via secreting special proteins, and subsequently recognized by host cells, ultimately triggering immune responses [30]. Hence, administration of ESA is often used to explore the underlying mechanism of maternal immune-activation-induced behavioral abnormalities [31] and maternal–fetal immune imbalance [32]. Regarding this, we successfully constructed a mouse abortion model to explore the underlying mechanism of *T. gondii*-induced adverse pregnancy outcomes.

Treg cells, as a vital subset of CD4^+^ T cells, are a vital regulator in maintaining maternal–fetal immune tolerance in humans and mice [33]. Treg cells can be divided into induced Treg cells (iTregs, also called pTreg) and naturally occurring Treg cells (nTregs). The latter comprises most of the Treg population in pregnant mice and expresses multiple immunosuppressive molecules, including glucocorticoid-induced TNFR-related protein (GITR) and cytotoxic T-lymphocyte-associated protein-4 (CTLA-4) [34]. Foxp3 is a specific transcriptional regulator necessary for the development, maintenance, and functional maturation of Tregs [35]. Although Foxp3^−^ Treg subsets enable to repress T cell proliferation via secreting and TGF-β and IL-10 in a cell-contact independent and dependent manner as well [36], nTregs with stable Foxp3 expression are identified as the most effector Tregs activated in the decidua. Compared with iTregs, Foxp3^+^ nTregs acquire high immunosuppressive capacity to maintain maternal–fetal immune tolerance. In humans, low levels of circulating Treg cells have been served as a predictive signal of a risk of miscarriage in pregnant women [37]. Previous studies have shown that infection of *T. gondii* in mice during pregnancy reduces the quantity and suppressive capacity of Foxp3^+^ Treg cells, suggesting that adverse outcomes triggered by *T. gondii* are partially due to dysfunction of Foxp3^+^ Treg cells [38].

microRNAs (miRNAs), which belong to a class of short endogenous non-coding RNA, are enabled to govern gene expression, especially at the post-transcriptional level via targeting the 3' UTR of messenger RNA (mRNA) [39]. Multiple miRNAs are shown to modulate cell development and function, which mainly inhibit the expressions of Foxp3 and other related genes via binding to mRNA 3′UTR. miR-133a-3p inhibits Foxp3 expression by binding to Foxp3 3′ UTR, thereby promoting proliferation and autophagy in gastric cancer cells [18]. miR-7 can directly bind to the 3′ UTR of T-cell acute lymphocytic leukemia protein 1 (TAL1) to suppress the migration, invasion, and cell motility of T-cell acute lymphoblastic leukemia cells, which can be reversed by TAL1 overexpression [40]. Nevertheless, there is evidence that miRNAs can stabilize translation complexes and promote protein production as well. Wang et al. found the increased miRNA-10a and miRNA-21 in CD4^+^CD25^+^ T cells compared with CD4^+^CD25^−^ T cells, indicating both of these miRNAs might heighten suppressor function of Treg cells via stabilizing Foxp3 expression [41]. Previous studies have established that miRNAs exhibit differential expression following *T. gondii* infection, and are influenced by the virulence of different strains [42]. This suggests that miRNAs have the potential to regulate *T. gondii* infection. Previous studies have also reported that the maternal–fetal interface of *T. gondii-*infected mice, particularly during the early stage, displayed reduced Foxp3 expression [43]. In our present study, there is a possible correlation between the downregulation of Foxp3 and the expression of miR-7b and miR-16 in ESA-stimulated EL4 cells in vitro. Regarding in vitro assays, miR-7b mimics inhibited Foxp3 expression, whereas its inhibitors promoted Foxp3 expression, indicating miR-7b negatively governed Foxp3. Therefore, miR-7b suppressed Foxp3 expression, but did not stabilize Foxp3 expression in the context of *T. gondii* infection. These findings hint at a possible association between *T. gondii* infection in mice and unfavorable pregnancy outcomes, which are marked by decreased Foxp3 levels and increased miRNA expression. Additionally, ESA enhances miR-7b promoter activity in EL4 cells by interacting with the −1599/−1199 and −392/+201 regions of the miR-7b promoter. These regions may contain TF-responsive elements that contribute to increased promoter activity of miR-7b.

PPARγ, belonging to the nuclear receptor superfamily as a vital ligand-activated transcription factor, is the most extensively studied [44, 45]. Recent research suggests that PPARγ binds to PPARγ response elements (PPREs) in the promoter region of target miRNAs to mediate miRNA transcription [46]. PPARγ directly binds to a specific PPRE in the miR-23 promoter region in macrophages (Mps), thereby facilitating miR-23 transcription [47]. PPARγ also induces transcription of PPRE-containing miR-145 and miR-329 promoters [48]. PPARγ upregulated miR-711 transcription via directly targeting the pre-miR-711 promoter via promoting Col7a1 gene transcription [49]. PPARγ overexpression significantly increases miR-381 promoter activity, thus inhibiting the wingless and int-1 (Wnt) signaling pathway downstream of miR-381 in human bone mesenchymal stromal cells (BMSC) [50]. However, several recent studies have indicated a negative correlation between PPARγ expression and the expression of certain miRNAs. Activated PPARγ inhibited miR-21-5p transcriptional activity by targeting the miR-21-5p promoter region, thereby inhibiting lipid droplet accumulation, oxidative stress, and hepatic inflammation in a mouse model of non-alcoholic steatohepatitis (NASH) [51]. Huang et al. found that PPARγ could directly bind to a putative PPRE site (−2262 to −2248 bp) of the murine miR-106a promoter to repress its transcription activity in cerebrovascular endothelial cells [52]. These data suggest that Foxp3 interacts with PPARγ in EL4 cells and that *T. gondii* antigens can inhibit the expression of Foxp3 and PPARγ. In our study, treatment with *T. gondii* antigens resulted in a significant increase in miR-7b promoter activity. This increase was dependent on PPARγ, which binds to specific regions of the miR-7b promoter (−1242/−1231 and −128/−117). We observed that *T. gondii* antigens reduced PPARγ expression and increased miR-7b transcription, suggesting that PPARγ is a negative regulator of miR-7b transcription rather than its positive regulator in EL4 cells. PPARγ has been found to directly bind to the miRNA promoter region to promote its transcription [47]. We hypothesized that the miR-7b promoter contains a PPARγ-binding site. Mutations in the PPARγ binding sites in the miR-7b promoter reduced the promoter activity of miR-7b induced by *T. gondii* antigens. Thus, *T. gondii* antigens influence miR-7b promoter activity via a PPARγ-dependent mechanism.

As for a vital transcription regulator, specificity protein 1 (Sp1) participates in the regulation of downstream gene expression. Recent bioinformatic analysis suggests that Foxp3 may not be a direct target gene of miR-7b. However, the possibility that miR-7b indirectly inhibited Foxp3 expression via other transcription factors cannot be ruled out. miR-7b has been found to inhibit Sp1. Guo et al. identified that miR-7b directly binds to the 3' UTR of Sp1, leading to the suppression of its expression. This in turn decreases the transcription of tumor suppressor p53-binding protein 1 (TP53BP1) and influences radiosensitivity in non-small cell lung cancer [53]. Metastasis-associated protein 2 (MTA2) silencing induces miR-7 to inhibit Sp1 expression via targeting its 3' UTR. Sp1 knockdown increased kallikrein-10 (KLK10) expression, indicating that the miR-7/Sp1 axis acts as a key regulator of MTA2, affecting both KLK10 expression and mobility in cervical cancer cells [54]. Transcription factors directly regulate Foxp3 gene expression by targeting its promoters [55]. Our previous study revealed that *T. gondii* antigens suppress the promoter activity of Foxp3 via an Sp1 dependency mechanism [18]. In this present study, our data further revealed that *T. gondii* antigens could facilitate miR-7b to inhibit Sp1 expression by targeting the Sp1 3' UTR, thereby inversely regulating Foxp3 expression.

**Fig. 8:**
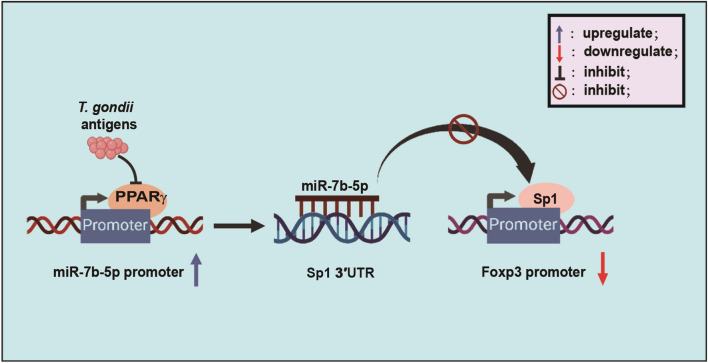
Schematic representation of Foxp3 regulation by miR-7b-5p. *T. gondii* antigens elevated the miR-7b-5p promoter activity by inhibiting PPARγ expression. Additionally, miR-7b-5p induced by *T. gondii* antigens directly targets Sp1 3' UTR to repress its expression, thereby suppressing Foxp3 promoter activity

Our study found that *T. gondii* infection results in an increase in miR-7b transcription but a decrease in Foxp3 expression. *T. gondii* antigens enhance miR-7b transcription by influencing promoter activity in a PPARγ-dependent mechanism. Despite not directly targeting the Foxp3 3' UTR, miR-7b can still inhibit Foxp3 expression by binding to the Sp1 3' UTR (Fig. 8). These findings suggest that Foxp3 expression can be regulated by direct gene regulatory regions and indirect regulation through miRNAs, particularly miR-7b. Understanding the relationship between miRNAs and *T. gondii*-induced Foxp3 expression can improve research on the treatment of *T. gondii*-related complications during pregnancy.

## Conclusions

Our current research has not only revealed an inverse correlation between the upregulation of miR-7b and the downregulation of Foxp3 in adverse pregnancy outcome caused by *T. gondii*, but also uncovered a potential molecular mechanism involving miR-7b-mediated regulation of Foxp3, which primarily affects the function of Treg cells. As more and more miRNAs interacting and synergizing with Foxp3 are identified, investigating miRNAs such as miR-7b in controlling Foxp3 expression will provide insights into immune-regulatory mechanisms that may be beneficial for the development of therapeutic strategies against *T. gondii* infection-induced pregnancy abnormalities.

Using tissue cysts to orally infect the mice is an appropriate way to construct the animal model of *T. gondii*-induced adverse pregnancy, which is one of the common sources of infection in nature. Both oral and intraperitoneal infections can lead to host immunity, and there may be some differences in the timing of triggering host immunity. However, there is currently no evidence to suggest any difference of two infections ways in host immune response, especially maternal–fetal immune tolerance. Currently, the intraperitoneal way to infect the mice is often being used for studies on the mechanism of abortion caused by *T. gondii* [56]*.*

### Supplementary Information

**Additional file 1: Figure S1.** SDS-PAGE analysis to show that ESA protein bands 12 A and B represent two biological replicates of the RH strain.

## Data Availability

Data are deposited in the website https://pan.baidu.com/s/1LaGwSBj30ZLE_B16syeD_A. The code is xb2l.

## Abbreviations

*T. gondii*: *Toxoplasma gondii*
Foxp3: Forkhead box P3
miRNAs: MicroRNAs
PPARγ: Peroxisome proliferator-activated receptor γ
Treg: Regulatory T
RPL: Recurrent pregnancy loss
PE: Preeclampsia
PTB: Preterm birth
RSA: Recurrent spontaneous abortion
SOCS1: Suppressor of cytokine signaling 1
RORγt: Receptor-related orphan receptor-gamma-t
ESA: Excreted-secreted antigens
Sp1: Specificity protein 1
dpi: Day post-infection
DMEM: Dulbecco’s modified Eagle’s medium
FBS: Fetal bovine serum
NC: Negative controls
WT: Wild type
MUT: Mutant
WB: Western blot
SDS-PAGE: Sodium dodecyl sulfate-polyacrylamide gel electrophoresis
PVDF: Polyvinylidene fluoride
ECL: Enhanced chemiluminescence
PCR: Polymerase chain reaction
Ct: Cycle time
SD: Standard deviation
ANOVA: Analysis of variance
De: Decidual zone
JZ: Junction zone
LZ: Labyrinth zone
TF: Transcription factor
ETS1: E26 transformation-specific-1
ELK1: Ets-like domain-containing protein 1
TLA: *T. gondii* Lysate antigens
iTregs: Induced Treg cells
nTregs: Naturally occurring Treg cells
GITR: Glucocorticoid-induced TNFR-related protein
CTLA-4: Cytotoxic T-lymphocyte-associated protein-4
mRNA: Messenger RNA
TAL1: T-cell acute lymphocytic leukemia protein 1
PPREs: PPARγ response elements
Mps: Macrophages
BMSC: Bone mesenchymal stromal cells
NASH: Non-alcoholic steatohepatitis
TP53BP1: Tumor suppressor p53-binding protein 1
MTA2: Metastasis-associated protein 2
KLK10: Kallikrein-10
