# Supplementary material for: Inhibition of Foxp3 expression in the placenta of mice infected intraperitoneally by toxoplasma gondii tachyzoites: insights into the PPARγ/miR-7b-5p/Sp1 signaling pathway
Source: Parasit Vectors. 2024 Apr 17;17:189. doi: 10.1186/s13071-024-06262-0 (PMC11025192; doi:10.1186/s13071-024-06262-0)
Supplement: Supplementary file 1 — Additional file 1: Figure S1. SDS-PAGE analysis to show that ESA protein bands 12 A and B represent two biological replicates of the RH strain. [file 13071_2024_6262_MOESM1_ESM.pdf]

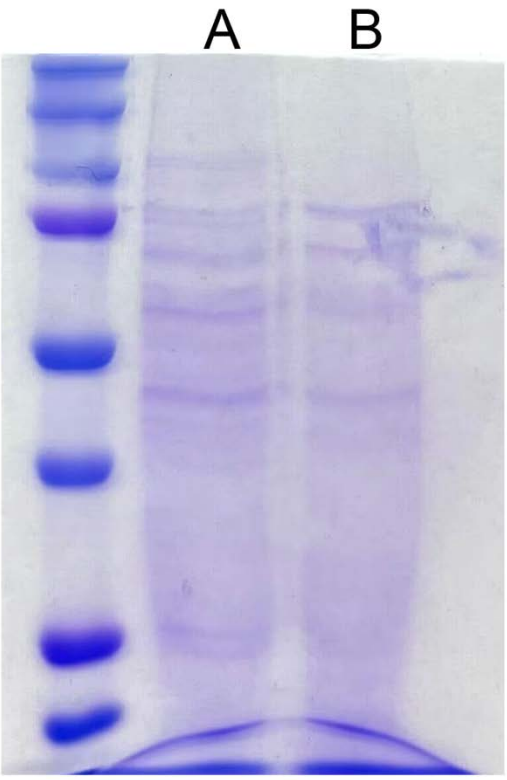

Fig. S1. SDS- PAGE analysis to show the ESA protein bands
A and B represent two biological replicates of the RH strain.

Yamamoto YI *et al* analyzed ESA by immunoblot assay and dot ELISA using serum samples from 25 patients with toxoplasmosis (19 in the acute phase and 6 chronic). They found that three groups of antigens, with molecular masses of 35, 30 32 and 26 kDa, reacted strongly with IgG antibodies, while antigens from two of these groups (30 32 and 26 kDa) also reacted with IgM and IgA [1].

References

1. Yamamoto YI, Mineo JR, Meneghisse CS, Guimarães AC, Kawarabayashi M. Detection in human sera of IgG, IgM and IgA to excreted/secreted antigens from Toxoplasma gondii by use of dot-ELISA and immunoblot assay. Ann Trop Med Parasitol. 1998; 92(1):23-30.
